# Regional insights on the prevalence and antimicrobial susceptibility pattern of carbapenem and colistin-resistant gram-negative bacteria: an observational cross-sectional study from Karachi, Pakistan

**DOI:** 10.1186/s12879-025-10535-z

**Published:** 2025-02-07

**Authors:** Nazia Khursheed, Fareeha Adnan, Moiz Ahmed Khan, Ramlla Hatif

**Affiliations:** Section of Microbiology, Department of Clinical Laboratory, Indus Hospital & Health Network, Plot C-76, Sector 31/5, Opposite Crossing, Darussalam Society, Sector 39 Korangi, Sindh, Karachi, Pakistan

**Keywords:** Colistin resistance, Carbapenem resistance, Multidrug resistance, Gram-negative bacteria, Antimicrobial susceptibility

## Abstract

**Background:**

Colistin is used to treat multidrug-resistant gram-negative bacteria. Rising colistin resistance worldwide has created challenges in effective treatment and raised treatment costs. Our study aimed to understand the prevalence of colistin resistance in carbapenem-resistant gram-negative bacteria, associated pathogens and their antimicrobial susceptibility pattern at our institute, to help limit further development of resistance.

**Methods:**

An observational, cross-sectional study was conducted at the Microbiology laboratory of Indus Hospital & Health Network, Karachi, Pakistan from January 1, 2022 to December 31, 2023. Variables extracted from the electronic patient care database included the type of patient samples, clinical diagnoses, frequency of carbapenem-resistant gram-negative bacteria and antimicrobial susceptibility profiles. Microsoft Excel software (Microsoft Excel 2013 {15.0.5553.1000} 32-bit) was used for analysis. Susceptibility results were interpreted in accordance with the M100 guidelines of the Clinical and Laboratory Standards Institute.

**Results:**

A total of 1,785 carbapenem-resistant gram-negative bacteria were isolated during the study period. Thirty (1.7%) of these exhibited colistin minimum inhibitory concentrations of ≥ 4 µg/ml and were characterized as colistin-resistant. Most patients with colistin-resistant gram-negative bacterial infections were males (57%), aged 31–49 and ≥ 50 years (37% each), and in-patients (60%). Majority had urinary tract infection (34%), followed by bloodstream infection (30%), ventilator-associated pneumonia (23%), and skin and soft tissue infection (13%). Organisms included *Klebsiella species* (77%), *Acinetobacter baumanii* (20%) and *Pseudomonas aeruginosa* (3%). Tigecycline was the most susceptible antibiotic among isolates (96%) while, fosfomycin (53%), minocycline (50%), doxycycline (45%) and tetracycline (42%) exhibited moderate susceptibility.

**Conclusion:**

Our study highlights a concerning prevalence of colistin resistance (1.7%) among carbapenem-resistant gram-negative bacteria, particularly *Klebsiella species*, predominantly affecting male in-patients aged 31–49 and ≥ 50 years. This significant therapeutic challenge is underscored by the limited efficacy of available antibiotics, with only tigecycline showing high susceptibility (96%) and others like fosfomycin (53%) and minocycline (50%) offering moderate alternatives.

## Introduction

Colistin is a polymyxin antibiotic that was commercialized in the 1950s and considered an effective treatment for most gram-negative organisms. However, its use was abandoned in 1980s in favor of other less toxic broad-spectrum antibiotics [1]. Rising antimicrobial resistance led to increased colistin use in 2000, and in 2007 the World Health Organization (WHO) re-classified polymyxins as major agents of choice for multidrug-resistant (MDR) gram-negative bacilli [2]. Amidst the global rise in incidence of hospital-acquired infections (HAIs) with MDR gram-negative bacteria such as *Pseudomonas aeruginosa*, *Acinetobacter baumanii*, *Klebsiella pneumoniae*, and other enterobacterales, concomitant colistin resistance has made treating these organisms increasingly challenging [3, 4]. Colistin is a last resort antibiotic for such infections and its overuse, particularly in intensive care units (ICUs), has led to the emergence of colistin-resistant gram-negative bacteria due to selective antibiotic pressure and horizontal transmission [3, 4]. Colistin-resistant gram-negative bacteria frequently exhibit carbapenem resistance as well and are categorized as extensively drug-resistant (XDR) [5]. Infections by colistin and carbapenem-resistant gram-negative bacteria (CCR-GNB) have been reported in many studies worldwide and present a significant challenge to appropriate treatment selection [4, 6, 7]. 

Initially, colistin-resistant strains were reported in the year 2000 in Europe [8, 9]. Monaco et al. found that 43% of carbapenemase-producing *K. pneumoniae* isolates were also resistant to colistin [10]. Colistin resistance has been observed in other enterobacterales, such as *E. coli*, and non-enterobacterales such as *Acinetobacter species* and *Pseudomonas aeruginosa* [7]. Southeast Asia has the highest rate of colistin resistance, followed by Europe and the United States of America. The prevalence of reported colistin resistance worldwide is less than 10% but is steadily on the rise [11]. According to a laboratory-based study from Pakistan, 15.9% enterobacterales were found to be colistin-resistant [12]. In another study, Asif et al. reported 3 colistin-resistant isolates, all of which were non-fermenters [13]. 

Colistin resistance is often related to higher mortality rates, longer hospital stay and higher treatment cost [3, 6, 7]. Factors that contribute to the development of colistin resistance mentioned in prior studies include past hospitalization, prolonged use of carbapenems and colistin, indwelling catheters, multiple co-morbidities and increasing age [14]. Antimicrobial alternatives against colistin-resistant gram-negative bacteria are limited. Emerging antimicrobials with adequate coverage against resistant infections are costly, making them inaccessible to resource-limited countries. Current treatment options for cases of CCR-GNB infections comprise of two or three drug combination regimens [6, 7, 15]. 

To effectively curb the development of colistin resistance, understanding its prevalence, associated clinical factors and variables driving its emergence is essential. Data on colistin resistance in Pakistan, however, remains limited. Our study uniquely addresses this gap by determining the prevalence of colistin resistance among CCR-GNB and identifying the associated pathogens and their antimicrobial susceptibility profiles at a tertiary care hospital in Karachi, Pakistan. Our objectives are to identify the incidence of these infections, characterize the demographic and clinical profiles of affected patients and evaluate the effectiveness of alternate antibiotics to help guide therapeutic strategies.

## Materials and methods

### Study setting and design

This observational, cross-sectional study was conducted at the Microbiology laboratory of Indus Hospital & Health Network in Karachi, Pakistan from January 1, 2022 to December 31, 2023. Indus Hospital & Health Network is one of the largest non-profit networks of primary, secondary and tertiary healthcare facilities across Pakistan.

As part of our institutional policy, colistin susceptibility is tested and reported in carbapenem-resistant gram-negative bacteria (CAR-GNB). All patient samples including blood, urine, tissue and respiratory specimens positive for CAR-GNB were included in the study via consecutive, non-probability sampling. Bacterial identification and antimicrobial susceptibility testing were performed after the clinical samples were processed for bacterial culture in the laboratory. After the bacterial strains were characterized as CAR-GNB, colistin susceptibilities were determined.

### Inclusion and exclusion criteria

All patient samples positive for gram-negative bacterial strains characterized as CAR-GNB received at the Indus Hospital Microbiology laboratory between January 1, 2022 to December 31, 2023, were included in the study. Patient samples were excluded from the study if (i) there was no bacterial growth or growth of bacteria other than gram-negative bacilli, (ii) the bacterial strains were characterized as carbapenem-susceptible or, (iii) they were received outside the pre-determined sample collection timeframe.

### Bacterial idntification and antimicrobial susceptiblity testing

Preliminary bacterial identification was done by gram stain and biochemical testing including catalase test, oxidase test, H_2_S production, indole test, hanging drop motility test, urease test, citrate test and triple sugar iron agar test. Identification was confirmed using API^®^ ID strips and *APIWEB*™ database (*bioMérieux*). Kirby-Bauer Disc Diffusion method was used to perform antibiotic susceptibility testing of isolates for antibiotics: amoxicillin-clavulanate (AMC), ampicillin (AMP), amikacin (AK), gentamicin (GN), ceftriaxone (CRO), cefixime (CFM), ciprofloxacin (CIP), levofloxacin (LEVO), piperacillin-tazobactam (TZP), imipenem (IPM), meropenem (MEM), trimethoprim/sulfamethoxazole (SXT), fosfomycin (FOT), tigecycline (TGC), ceftazidime (CAZ), doxycycline (DOX), tetracycline (TE), minocycline (MH) and cefoperazone/sulbactam (SCF). Zone diameter interpretation to label antibiotic activity as sensitive, intermediate or resistant, was done in accordance with antimicrobial susceptibility breakpoints mentioned in the Clinical & Laboratory Standards Institute (CLSI) M100 [16]. 

### Colisitin suceptibility testing via broth microdilution method

All CAR-GNB isolates were sub-cultured on Sheep Blood Agar (SBA) and incubated for 24 h in ambient air at 37 °C to perform colistin susceptibility testing via Broth Microdilution method (BMD). According to our laboratory protocol, isolated bacterial colonies were inoculated in phosphate-buffered saline to achieve a turbidity index of 0.5 McFarland. For BMD, 96-well U-bottom plates (Thermo Fisher) with doubling dilutions of colistin were used. Cation-adjusted Mueller Hinton broth (Sigma-Aldrich) was dispensed in wells followed with inoculation of bacterial suspension to obtain a final inoculum of 5 × 10^5^ CFU/mL in each well. The procedure was performed in accordance with the CLSI M7-A10 guidelines [17]. To assure quality control of bacterial growth and a visual comparison of bacterial inhibition in the antibiotic-dispensed wells, an equal concentration of media broth and bacterial suspension were dispensed in a growth control well without the antibiotic. Bacterial suspension for each isolate was also inoculated on 5% SBA to assess isolate purity and results were reported only when isolate purity was confirmed.

Minimum Inhibitory Concentration (MIC) of colistin was assessed by visual inspection for bacterial growth inhibition and determined based on the first well demonstrating growth inhibition. The quality of MIC values was evaluated by processing *Escherichia coli* ATCC strain 25922 with the isolates, and all results were considered accurate only when the MIC of the ATCC strain was within the acceptable range. All results were interpreted in accordance with the CLSI M100 guidelines [16]. Isolates exhibiting MICs of ≤ 2 µg/ml were considered intermediate whereas, those with MICs of ≥ 4 µg/ml were characterized resistant.

### Sample size calculation

Sample size was determined from the findings of a previous study from Pakistan evaluating the prevalence of colistin resistance among carbapenem-resistant enterobacterales (CRE), using the WHO sample size software [12, 18]. We estimated that a minimum sample size of 1,778 CAR-GNB strains is required to detect an expected colistin resistance of 15.9%, for a 95% confidence interval and 1.7% margin of error.

$$\begin{array}{l}\:n=\frac{{Z}^{2}\:p\left(1-p\right)}{{d}^{2}}\\=\:\frac{{\left(1.96\right)}^{2\:}\times\:0.159\times\:(1-0.159)}{{\left(0.017\right)}^{2}}\\=\:\text{1,778}\:isolates\end{array}$$

*n = Sample size*,* Z = Z statistic for a level of confidence (Z = 1.96 for a confidence level of 95%)*,

*p = Expected prevalence or proportion and d = Precision.*

### Data collection and statistical analysis

Baseline characteristics, demographic and clinical data of the study population was retrieved from the electronic patient care database and documented on a standardized paper-based questionnaire. Data was analyzed after removing patient identifiers to ensure confidentiality and anonymity. Variables recorded included patient age, gender, admitting department, type of clinical samples, clinical diagnoses, frequency of CAR-GNB isolates and susceptibility to colistin and other antibiotics. Data collected on study questionnaires was entered into Microsoft Excel software (Microsoft Excel 2013 {15.0.5553.1000} 32-bit) for analysis. Data entry verification during this transfer process was performed twice for each variable. Chi-squared test was used to identify significant associations between the demographic characteristics of the patient population and the frequency of CCR-GNB infections. Statistical association was also assessed between the type of colistin-resistant organisms with specific colistin MIC values. A *p* value of ≤ 0.05 was considered “significant”.

## Results

During the study period, a total of 11,900 gram-negative bacterial infections were reported, of which 1,785 (15%) were CAR-GNB strains. Thirty (1.7%) of these exhibited colistin MICs of ≥ 4 µg/ml and were characterized as resistant. Majority of patients with colistin-resistant gram-negative bacterial infections were males (57%) between the ages of 31–49 and ≥ 50 years (37% each), and in the in-patient setting (60%). No statistical significance was found (Table 1).

**Table 1:** Demographics of patients with colistin-resistant gram-negative bacterial infections

Patient characteristics	*n* (%)	*p* value
Age (years)		
0–30 31–49 ≥ 50	8 (26) 11 (37) 11 (37)	*p* = 0.6
		*p* = 0.8
		*p* = 0.8
Gender		
Male	17 (57)	*p* = 0.9
Female	13 (43)	*p* = 0.9
Patient Location		
In-patient	18 (60)	*p* = 0.8
Out-patient^a^	7 (23)	*p* = 0.6
Emergency	5 (17)	*p* = 0.5

^a^ All patient encounters other than those admitted to the hospital or in the emergency department, including outpatient clinics

Majority of CCR-GNB strains were isolated from urine samples (*n* = 10; 34%), and most patients had urinary tract infection (UTI) (*n* = 10; 34%), of which two were diagnosed with catheter-associated urinary tract infection (CAUTI) (Table 2).

**Table 2:** Types of infection and sample in patients with colistin-resistant Gram-negative bacterial infections

Sample Type	*n* (%)
Urine	10 (34)
Blood	8 (27)
Tracheal Aspirate	6 (20)
Pus	4 (13)
Sputum	1(3)
Central Venous Line Tip	1(3)
Infection Type ^a^	n(%)
UTI	10 (34)
CAUTI	2 (20)
BSI	9 (30)
CLABSI	2 (22)
VAP	7 (23)
SSTI	4 (13)

^a^ UTI: urinary tract infection, CAUTI: catheter-associated urinary tract infection, BSI: bloodstream infection, CLABSI: central line-associated bloodstream infection, VAP: ventilator-associated pneumonia, SSTI: skin and soft tissue infection

Pathogens exhibiting CCR-GNB strains were most commonly Enterobacterales (*n* = 23; 77%), all of which were *Klebsiella species*, followed by *Acinetobacter baumanii* (*n* = 6; 20%) and *Pseudomonas aeruginosa* (*n* = 1; 3%).

**Fig. 1:**
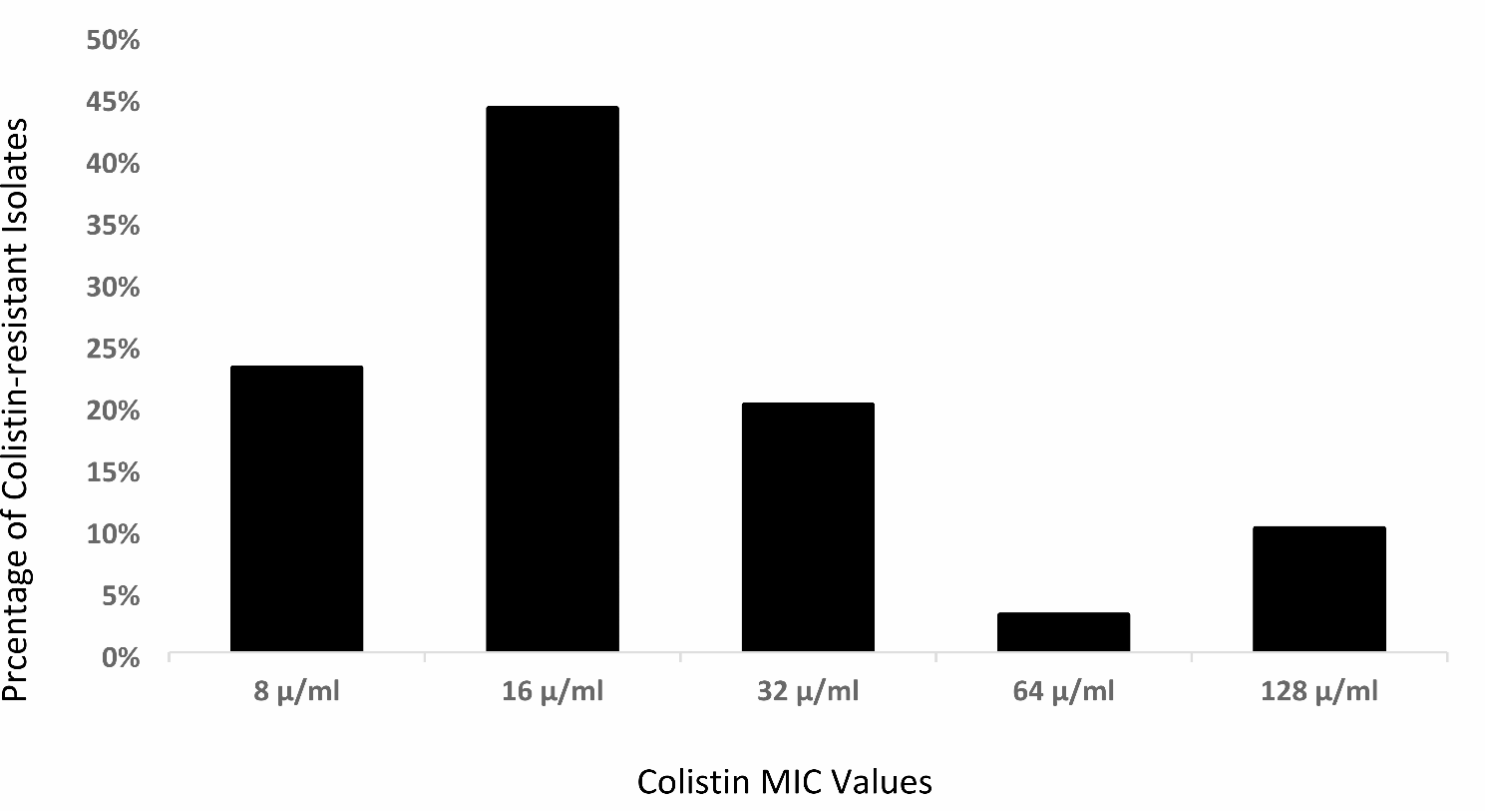
Distribution of colistin MICs among carbapenem and colistin-resistant gram-negative bacteria

The distribution of colistin MICs among CCR-GNB isolates is shown in Fig. 1. Among *Klebsiella species*, majority had an MIC of 16 µg/ml (*p* = 0.38). However, there was no statistically significant association between the colistin-resistant organisms and any specific value of colistin MIC (Table 3).

**Table 3:** Colistin MIC values among different carbapenem-resistant gram-negative bacteria (statistical significance: *p* = ≤ 0.05)

MIC µ/ml (*n*)	Organisms *n* (%) *p*-value		
	*Klebsiella species* (*n* = 23)	*Acinetobacter baumanii* (*n* = 6)	*Pseudomonas aeruginosa* (*n* = 1)
8 (7)	6 (26) *p* = 0.50	1 (1) *p* = 0.67	0 (0)
16 (13)	11 (48) *p* = 0.38	2 (33) *p* = 0.58	0 (0)
32 (6)	5 (22) *p* = 0.67	1 (17) *p* = 0.80	0 (0)
64 (1)	1 (4) *p* = 1.00	0 (0)	0 (0)
128 (3)	0 (0)	2 (33) *p* = 0.07	1 (100) *p* = 0.40

**Fig. 2:**
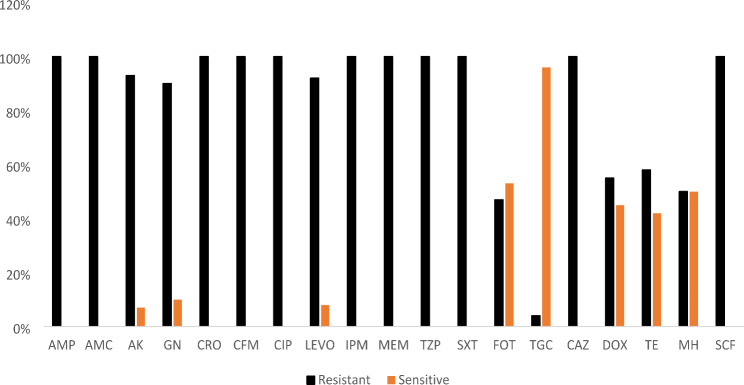
Antimicrobial susceptibility profile of CCR-GNB isolates

The antimicrobial susceptibility patterns showed most isolates were sensitive to TGC (96%), and moderately sensitive to FOT (53%), MH (50%), DOX (45%) and TE (42%). Least susceptibility was observed to GN (10%), LEVO (8%) and AK (7%), and none to AMC, AMP, CRO, CFM, CAZ, CIP, SXT, SCF, TZP, IPM and MEM (Fig. 2).

## Discussion

The emergence of MDR and XDR microorganisms presents a significant challenge to appropriate antibiotic selection worldwide. Countries with limited healthcare resources, in particular, struggle to manage patients with MDR bacterial infections, leading to higher morbidity and mortality rates. Colistin has been used in the past to treat resistant gram-negative bacterial infections, but overuse of this antibiotic has led to the emergence of colistin-resistant gram-negative bacterial infections, which are also frequently resistant to carbapenem antibiotics [3–5]. During our two-year study period, 1.7% colistin resistance was observed among CAR-GNB isolates. Our findings are similar to a study from China conducted on 1,868 CRE isolates between 2014 and 2019, that showed a prevalence of 0.41% before and 1.38% after colistin was introduced into clinical practice. Increase in CCR-GNB isolates mirrored the pattern of colistin use following its official release in 2017 [19]. 

Increased clinical use of colistin as a last resort therapy for CAR-GNB infections has followed an increase in the use of carbapenem drugs. Recent studies from South Asia have reported significant increase in colistin resistance. A study from Punjab, Pakistan reported 10.5% colistin resistance among 172 CRE isolates tested using the Colistin agar test [20]. Another study conducted in Khyber Pakhtunkhwa, Pakistan reported an even higher prevalence of colistin resistance (19.9%) among the 241 uropathogenic gram-negative bacterial strains tested using BMD [21]. A study from New Delhi, India reported 15% colistin resistance among 100 CRE isolates [22].

Most patients with CCR-GNB infections in our study had UTI (34%), followed by bloodstream infection (BSI) (30%). Several other studies have found UTI to be the most prevalent infection caused by CCR-GNB, including Arjun et al. who found 33% isolates in urine samples [6, 14, 17, 23]. Zafar et al. found the highest frequency of colistin-resistant MDR *E. coli* and *K. pneumoniae* strains from cancer patients in BSI, similar to that reported by Capone et al. (24–25) The latter included patients from ICU, who often have central venous access, which predisposes them to central line-associated bloodstream infections (CLABSI). In contrast, Syed et al. found ventilator-associated pneumonia (VAP) to be the most common infection caused by CCR-GNB in the ICU [26]. Since our study included both in-patient and out-patient samples and was not limited to the ICU, the incidence of VAP and CLABSI may have differed from that reported in these studies.

Similar to other studies from South Asia, Europe and Brazil, *Klebsiella species* were the most prevalent in our study, in contrast to the findings of Ramesh et al. and Manohar et al., who reported *E. coli* as the most common [15, 23, 27–30]. Similar to Rossi et al., our isolates also included non-enterobacterales, such as *Acinetobacter baumanii* (*n* = 6) and *Pseudomonas aeruginosa* (*n* = 1) [28]. *Acinetobacter species* have been reported as the most frequent CCR-GNB in some studies [8, 29, 30]. The isolates in our study are mentioned in the WHO’s list of critical and high priority pathogens, further emphasizing the need to monitor the development of resistance in these organisms [31]. Mutations in chromosomes (e.g. *mgrB*,* phoP/ phoQ*,* and pmrA/ pmrB*) and acquisition of plasmids like *mcr-1*, modifies lipid A, reduces affinity for colistin and contributes to resistance [32]. 

The antimicrobial susceptibility profile of our isolates showed that TGC was the most susceptible, similar to the findings of Elnasser et al., who reported that TGC exhibited relatively higher susceptibility (78.6%) compared to SXT (32%), TE (22%), aztreonam (8.6%), and cefepime (5.3%) [33]. Another study from Karachi, Pakistan of 93 CCR-GNB strains reported FOT (72%) and TGC (50.5%) to be the most susceptible antibiotics [26]. Zhou et al. also observed that when colistin and tigecycline (TGC) were used in combination at clinically relevant concentrations, they significantly reduced the bacterial density of colistin-resistant *E. coli*. A concentration-dependent reduction in colistin MIC was also noted as TGC levels increased. These findings suggest that the combination of these drugs could serve as a therapeutic alternative for infections caused by MDR *E. coli* harboring the blaNDM-5 and mcr-1 genes [34]. Antimicrobial resistance is on the rise in Pakistan, driven by challenges at multiple levels of the healthcare system. In cases where health regulatory bodies have not developed a strong framework for training and overseeing the safe use of antibiotics, physicians often bear responsibility for inappropriate prescribing. Additionally, the issue is exacerbated by the widespread lack of patient education on proper antibiotic use and misuse, along with common self-medication practices. This problem could be significantly alleviated through strict government regulations, a code of conduct for physicians prescribing antibiotics, ethical marketing practices, and a commitment to prescription ethics.

## Conclusion

Our study demonstrates the presence of colistin resistance in carbapenem-resistant gram-negative bacteria, presenting a significant therapeutic challenge due to limited availability of alternative antimicrobials. Increasing resistance rates highlight the need for surveillance of antibiotic susceptibility data, development of institutional antibiograms to guide treatment and a strong emphasis on antimicrobial stewardship. We recommend a multi-center study to investigate patient characteristics associated with the development of colistin-resistant antimicrobial strains, which could help guide appropriate treatment selection in resource-limited settings.

### Limitations

There were several limitations in our study. As this was not a prospective, observational study we were unable to analyze long-term patient outcomes. We were also unable to describe patient factors including specific patient location in the hospital (ward versus ICU), length of hospital stay and, type and duration of antibiotic therapy administered. These clinically relevant factors are important in understanding the development of colistin-resistant infections. Due to cost restraints, we were unable to use molecular diagnostics to analyze genetic determinants of resistance which would have further strengthened our conclusions. Lastly, this was a single-center study. We recommend a multi-center study to understand the true prevalence of colistin-resistant infections across diverse healthcare settings in Pakistan and other parts of the world. Such a study will also allow for stronger generalizability of findings. Addressing these limitations in future research will help in gaining a comprehensive understanding of colistin resistance and improving patient outcomes.

## Data Availability

All data generated and analyzed during the course of the study is mentioned in this article.

## References

[1] Hamel M, Rolain J-M, Baron SA. The history of colistin resistance mechanisms in bacteria: Progress and challenges. Microorganisms. 2021;9(2):442. Available from: 10.3390/microorganisms9020442 10.3390/microorganisms9020442 PMC7924381 33672663

[2] WHO List of Medically Important Antimicrobials. who.int. [cited 2024 Dec 5]. Available from: https://cdn.who.int/media/docs/default-source/gcp/who-mia-list-2024-lv.pdf 10.1016/S1473-3099(24)00248-2 38677312

[3] Kontopidou F, Plachouras D, Papadomichelakis E et al. Colonization and infection by colistin-resistant Gram-negative bacteria in a cohort of critically ill patients. Clin Microbiol Infect. 2011;17(11):E9–11. Available from: 10.1111/j.1469-0691.2011.03649.x 10.1111/j.1469-0691.2011.03649.x 21939468

[4] Marchaim D, Chopra T, Pogue JM. Outbreak of colistinresistant, carbapenem-resistant Klebsiella pneumoniae in metropolitan Detroit, Michigan. Antimicrob Agents Chemother. 2011;55(2):593–9. 21115786 10.1128/AAC.01020-10 PMC3028794

[5] Magiorakos A-P, Srinivasan A, Carey RB et al. Multidrug-resistant, extensively drug-resistant and pandrug-resistant bacteria: an international expert proposal for interim standard definitions for acquired resistance. Clin Microbiol Infect. 2012;18(3):268–81. Available from: 10.1111/j.1469-0691.2011.03570.x 10.1111/j.1469-0691.2011.03570.x 21793988

[6] Paterson DL, Harris PNA. Colistin resistance: a major breach in our last line of defence. Lancet Infect Dis. 2016;16(2):132–3. Available from: 10.1016/S1473-3099(15)00463-6 10.1016/S1473-3099(15)00463-6 26603171

[7] Olaitan AO, Morand S, Rolain JM. Emergence of colistin-resistant bacteria in humans without colistin usage: a new worry and cause for vigilance. Int J Antimicrob Agents. 2016;47(1):1–3. 26712133 10.1016/j.ijantimicag.2015.11.009

[8] Valero E, Sevillano D, Calvo A, et al. Activity of new fluoroquinolones against clinical isolates of Acinetobacter baumannii. Rev Esp Quimioter. 2001;14(4):358–63. 11856982

[9] Gales AC, Jones RN, Sader HS. Global assessment of the antimicrobial activity of polymyxin B against 54 731 clinical isolates of Gram-negative bacilli: report from the SENTRY antimicrobial surveillance programme (2001–2004). Clin Microbiol Infect. 2006;12(4):315–21. Available from: 10.1111/j.1469-0691.2005.01351.x 10.1111/j.1469-0691.2005.01351.x 16524407

[10] Monaco M, Giani T, Raffone M et al. Colistin resistance superimposed to endemic carbapenem-resistant Klebsiella pneumoniae: a rapidly evolving problem in Italy, November 2013 to April 2014. Euro Surveill. 2014;19(42). Available from: 10.2807/1560-7917.es2014.19.42.20939 10.2807/1560-7917.es2014.19.42.20939 25358041

[11] Bialvaei AZ, Samadi Kafil H. Colistin, mechanisms and prevalence of resistance. Curr Med Res Opin. 2015;31(4):707–21. Available from: 10.1185/03007995.2015.1018989 10.1185/03007995.2015.1018989 25697677

[12] Qamar S, Shaheen N, Shakoor S et al. Frequency of colistin and fosfomycin resistance in carbapenem-resistant Enterobacteriaceae from a tertiary care hospital in Karachi. Infect Drug Resist. 2017;10:231–6. Available from: 10.2147/IDR.S136777 10.2147/IDR.S136777 PMC5546765 28814888

[13] Asif A, Cheema KH, Ashraf Z. Colistin resistance among gram-negative non fermentors isolated from patients at a tertiary care referral burn center. Pak J Pathol. 2018;29:4–7.

[14] Richter SE, Miller L, Uslan DZ et al. Risk factors for colistin resistance among Gram-negative rods and Klebsiella pneumoniae isolates. J Clin Microbiol. 2018;56(9):JCM.00149– 18. Available from: 10.1128/jcm.00149-18 10.1128/JCM.00149-18 PMC6113453 29976595

[15] Suh J-Y, Son JS, Chung DR et al. Nonclonal emergence of colistin-resistant Klebsiella pneumoniae isolates from blood samples in South Korea. Antimicrob Agents Chemother. 2010;54(1):560–2. Available from: 10.1128/AAC.00762-09 10.1128/AAC.00762-09 PMC2798536 19752282

[16] Clinical & Laboratory Standards Institute M100 Ed. 34 [cited 2024 Aug 22]. Available from: https://clsi.org/standards/products/microbiology/documents/m100/

[17] Clinical & Laboratory Standards Institute M07. Ed12 [cited 2024 Nov 6]. Available from: https://clsi.org/standards/products/microbiology/documents/m07/

[18] WHO Sample Size Calculator. Haematologywatch.net. [cited 2024 Dec 3]. Available from: https://haematologywatch.net/sample-size-calculator.php

[19] Huang H, Dong N, Shu L et al. Colistin-resistance gene *mcr* in clinical carbapenem-resistant *Enterobacteriaceae* strains in China, 2014–2019. Emerg Microbes Infect. 2020;9(1):237–45. Available from: 10.1080/22221751.2020.1717380 10.1080/22221751.2020.1717380 PMC7034111 31996107

[20] Furqan W, Ali S, Usman J et al. Assessing colistin resistance by phenotypic and molecular methods in carbapenem-resistant Enterobacterales in a tertiary care hospital in Pakistan. Infect Drug Resist. 2022;15:5899–904. Available from: 10.2147/idr.s376490 10.2147/IDR.S376490 PMC9553232 36237291

[21] Arif A, Ullah I, Ullah O et al. Identification of colistin resistance and its bactericidal activity against uropathogenic gram negative bacteria from Hayatabad Medical Complex Peshawar. Pak J Med Sci Q. 2022;38(4). Available from: 10.12669/pjms.38.4.5221 10.12669/pjms.38.4.5221 PMC9121951 35634614

[22] Bir R, Gautam H, Arif N et al. Analysis of colistin resistance in carbapenem-resistant *Enterobacterales* and XDR *Klebsiella pneumoniae*. Ther Adv Infect Dis. 2022;9:204993612210806. Available from: 10.1177/20499361221080650 10.1177/20499361221080650 PMC8883296 35237435

[23] Arjun R, Nambi PS, Kumar DS et al. A study of 24 patients with colistin-resistant Gram-negative isolates in a tertiary care hospital in South India. Indian J Crit Care Med. 2017;21(5):317–21. Available from: 10.4103/ijccm.ijccm_454_16 10.4103/ijccm.IJCCM_454_16 PMC5455025 28584435

[24] Zafer MM, El-Mahallawy HA, Abdulhak A et al. Emergence of colistin resistance in multidrug-resistant Klebsiella pneumoniae and Escherichia coli strains isolated from cancer patients. Ann Clin Microbiol Antimicrob. 2019;18(1):40. Available from: 10.1186/s12941-019-0339-4 10.1186/s12941-019-0339-4 PMC6909591 31831019

[25] Capone A, Giannella M, Fortini D et al. High rate of colistin resistance among patients with carbapenem-resistant Klebsiella pneumoniae infection accounts for an excess of mortality. Clin Microbiol Infect. 2013;19(1):E23–30. Available from: 10.1111/1469-0691.12070 10.1111/1469-0691.12070 23137235

[26] Syed B, Ishaque S, Imran A et al. Emergence of colistin-resistant gram-negative rods in intensive care units: A cross-sectional study from a developing country. SAGE Open Med. 2022;10:205031212211323. Available from: 10.1177/20503121221132358 10.1177/20503121221132358 PMC9583228 36277441

[27] Giani T, Pini B, Arena F et al. Epidemic diffusion of KPC carbapenemase-producing Klebsiella pneumoniae in Italy: results of the first countrywide survey, 15 May to 30 June 2011. Euro Surveill. 2013;18(22). Available from: 10.2807/ese.18.22.20489-en 23787077

[28] Rossi F, Girardello R, Cury AP et al. Emergence of colistin resistance in the largest university hospital complex of São Paulo, Brazil, over five years. Braz J Infect Dis. 2017;21(1):98–101. Available from: 10.1016/j.bjid.2016.09.011 10.1016/j.bjid.2016.09.011 PMC9425531 27832961

[29] Ramesh N, Prasanth M, Ramkumar S, Suresh M, Tamhankar AJ, Gothandam KM, et al. Colistin susceptibility of gram-negative clinical isolates from Tamil Nadu. Asian Biomed. 2016;10(1):35–9. https://intapi.sciendo.com/pdf/10.5372/1905-7415.1001.462

[30] Manohar P, Shanthini T, Ayyanar R et al. The distribution of carbapenem- and colistin-resistance in Gram-negative bacteria from the Tamil Nadu region in India. J Med Microbiol. 2017;66(7):874–83. Available from: 10.1099/jmm.0.000508 10.1099/jmm.0.000508 28671537

[31] WHO bacterial priority pathogens list. 2024: Bacterial pathogens of public health importance to guide research, development and strategies to prevent and control antimicrobial resistance. Who.int. World Health Organization; 2024 [cited 2024 Oct 19]. Available from: https://www.who.int/publications/i/item/9789240093461

[32] Liu Y-Y, Wang Y, Walsh TR, Yi L-X, Zhang R, Spencer J et al. Emergence of plasmid-mediated colistin resistance mechanism MCR-1 in animals and human beings in China: a microbiological and molecular biological study. Lancet Infect Dis. 2016;16(2):161–8. Available from: 10.1016/S1473-3099(15)00424-7 10.1016/S1473-3099(15)00424-7 26603172

[33] Elnasser Z, Elsamarneh R, Obeidat H et al. In-vitro activity of tigecycline against multidrug-resistant Gram-negative bacteria: The experience of a university hospital. J Infect Public Health. 2021;14(4):478–83. Available from: 10.1016/j.jiph.2020.12.013 10.1016/j.jiph.2020.12.013 33743369

[34] Zhou Y-F, Liu P, Zhang C-J et al. Colistin combined with tigecycline: A promising alternative strategy to combat Escherichia coli harboring bla NDM- 5 and mcr-1. Front Microbiol. 2019;10:2957. Available from: 10.3389/fmicb.2019.02957 10.3389/fmicb.2019.02957 PMC6960404 31969868

